# Impact of Body Reserves Dynamic on Productivity and Reproductive Performance in Fat-Tail and Thin-Tail Sheep Breeds over Contrasting Production Cycles

**DOI:** 10.3390/ani14060891

**Published:** 2024-03-14

**Authors:** Yathreb Yagoubi, Samir Smeti, Mokhtar Mahouachi, Massara Nasraoui, Samia Ben Saïd, Aziza Mohamed-Brahmi, Naziha Atti

**Affiliations:** 1 Laboratoire des Production Animale et Fourragère, Institut National de la Recherche Agronomique de Tunisie, University of Carthage, Rue Hedi Karray, Ariana 2049, Tunisia; 2 Appui à la Durabilité des Systèmes de Production Agricoles du Nord-Ouest, Ecole Supérieure d’Agriculture du Kef, University of Jendouba, Le Kef 7119, Tunisia

**Keywords:** ewes, body condition score, body reserves, fertility, lamb’s growth

## Abstract

**Simple Summary:**

Climate change presents significant nutritional challenges to animal breeding, demanding resilience and efficiency for survival and productivity. In this study, we investigated the effects of body reserves dynamics (mobilization and accretion) on the production and reproduction parameters of fat-tailed Barbarine and thin-tailed Queue Fine de l’Ouest ewes over two production cycles under semi-intensive conditions. The results show that all reproductive traits were similar among breeds; however, they depend on ewes’ body reserves. Similarly, lambs’ growth parameters depend on ewes’ body condition scores (BCSs) at lambing and weaning, with higher growth for ewes with BCS > 2 at lambing. In conclusion, regardless of the breed, ewes have evolved adaptive mechanisms by mobilizing body reserves to effectively cope with environmental challenges and successfully rear their offspring. Additionally, to sustain a high growth rate in offspring, it is advisable to carefully plan the flock nutritional needs during the suckling period.

**Abstract:**

Under climate change, animal breeding faces severe nutritional challenges, exhibiting their resilience and efficiency to survive and produce. The animals’ robustness can be evaluated through the use and reconstitution of body reserves (BR). This study investigated the dynamics of ewes’ BR, measured through body weight (BW) and the body condition score (BCS), and their effect on production performances of 480 ewes belonging to fat-tailed Barbarine (BB) and thin-tailed Queue Fine de l’Ouest (QFO) ewes over two consecutive years. The BW and BCS were recorded across physiological stages. Reproductive parameters and lambs’ growth were calculated. For both years and breeds, the decrease in the BCS between mating and lambing showed BR mobilization to balance nutritional restrictions, which continues until suckling; after weaning, a BR accretion was noted. The lambs’ birth weight was similar regardless of the dams’ BCS at lambing, though it was lower in the second year (3.5 vs. 4 kg). Lambs’ growth parameters depended on ewes’ BCS at lambing and weaning. Fertility rates changed with BCS changes and the higher values (80%) were recorded for ewes with BCSs between 2 and 2.5. However, prolificacy was similar for both breeds and years (*p* > 0.05). In conclusion, ewes have developed adaptative capacities through BR mobilization to cope with environmental challenges and successfully rear their offspring. To maintain a high offspring growth rate, it is recommended to plan correct flock nutrition during suckling.

## 1. Introduction

Small ruminants have an important role on social, economic, ecological, and cultural levels in most parts of the world. However, they face the challenges of frequent climatic perturbation associated with climate change, continuous rising costs, and seasonal feed restrictions [1,2,3]. In such situations, sheep production needs to improve its ability to adapt to the changing production conditions, primarily by breeding robust and resilient animals [4,5,6]. Hence, in the future, breeding strategies will include robust animals able to adapt to variations in feed availability [7,8]. There are several descriptions and explanations of resilience and robustness; they mainly result in animals that combine productivity and an adaptive capacity to harsh conditions [2,9,10]. In semi-arid conditions such as the Mediterranean South, sheep are often submitted to food shortages and severe climate conditions during several months per year. In many cases, these difficulties coincide with high energy-demanding periods (late pregnancy–early lactation) that cannot be fully compensated for by feed intake only [11,12]. In periods of feed restriction, ewes use their body reserves (BR) stored in fat and muscle tissue [13] to balance the nutritional requirements for maintenance, production, and reproduction [14,15]. This is especially true during dry seasons, when the feed availability is low. Later, when food availability increases again, BR are reconstituted. The dynamics of BR expresses the capacity of ruminants to efficiently adapt to feed intake and characterize the resilience/robustness of the individuals [2,8,16].

The most commonly used method to assess the nutritional status, BR, and their dynamics for an animal is its body weight (BW), given that it is easier and quicker to perform and does not require expertise [17]. However, for the past several years, BR have been measured using body condition scoring (BCS) as the most accurate method, given that the body weight alone cannot indicate the degree of fat and the parts of the body mass and the digesta [18]. BCS is a subjective measure based on the palpation of subcutaneous fat and muscle of the lumbar region [19].

BCS has been widely adopted to manage the nutritional strategy of flocks based on the adaptive capacities of animals in a given environment. Hence, many researchers have studied the effects of body weight and BCS around mating or parturition on productive and reproductive traits under different rearing systems [2,10,20]. 

Many studies have measured the impact of BR dynamics in the reproduction and productivity of thin-tail breeds during critical periods. However, to our knowledge, there are only few studies concerning sheep productivity associated with BR dynamics in fat-tail sheep breeds; these breeds have a supplementary site of BR at the tail level. Therefore, the present study aimed to assess the productive and reproductive performances of Barbarine ewes, a fat-tail breed, compared to Queue Fine de l’Ouest ewes, a thin-tail breed, in relationship with their BR dynamics (mobilization and accretion) through two successive and fluctuating production cycles, under semi-arid environmental conditions, and using BW and the BCS as monitor tools.

## 2. Material and Methods

### 2.1. Experimental Location, Animals, and General Management

**Figure 1:**
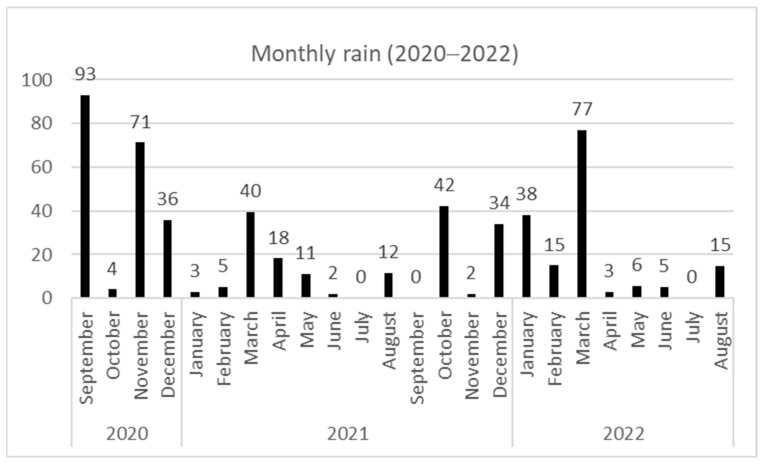
Average monthly rainfall of two campaigns (2020–2022).

The study was accomplished over two consecutive years (2020–2022) in the farm of the “Livestock and Pasture Office” in Saouef, located in Zaghouan province of the Tunisian North (36°13′44″ N 10°10′18″ E; Altitude = 167 m). This area belongs to the semi-arid bioclimatic stage characterized by a hot summer and large inter- and intra-year rainfall variability. Figure 1 reports the average monthly rainfall for both experimental years (meteorologic station of Saouef). The annual precipitation for the first year (2020–2021) was 294 mm with 42 rainy days; however, for the second year (2021–2022), a decrease in rainfall by 20% was recorded (236 mm), with only 36 rainy days. 

The study was performed on three flocks belonging to the main sheep breeds in Tunisia, including two flocks of the Barbarine breed (BB) and one of the Queue Fine de l’Ouest breed (QFO). Both breeds have an adult BW of 60–70 kg for males and 40–50 kg for females, their first mating is carried out at 18 months of age, and their first lambing is obtained at 24 months of age.

All flocks were averagely composed of 160 ewes aged from 2 to 6 years, with 84% multiparous and 16% primiparous. All flocks were kept under similar management conditions from mating to weaning. They were managed under a semi-intensive system, where they were kept indoors during the night and grazed during the day (6–7 h/day) on natural range lands that were fallow and/or stubble throughout the year except during winter or during feed-shortage periods. In combination with grazing and to fulfill their nutritional needs, the animals received hay, straw, cactus, some fodder shrubs (acacia), and concentrate according to their requirements [21]. The concentrate offered to the animals was either commercially produced, home-produced, or a combination of commercially and home-produced.

### 2.2. Reproductive Management

The reproduction management was based on a once-a-year mating period during spring using the ram effect, and therefore lambing occurred in autumn. The breeding season of sheep in this location extended from late April until late July, and thus ewes started lambing in late September–early October and milked their lambs for 5 to 6 months. For fat-tailed Barbarine flocks, estrus behavior was monitored daily by the shepherd, who assisted the ram in mating with the ewe. Estrus took place in the shade either in the early morning or late afternoon at cool temperatures. For each mated ewe from BB breed, the date and the ram number were registered; however, for the QFO ewes, none of these data was recorded, as the mating for this breed is not assisted. In the last third of pregnancy, all ewes received a supplement of 250–300 g/d of concentrate. 

### 2.3. Measurements on Animals

**Figure 2:**
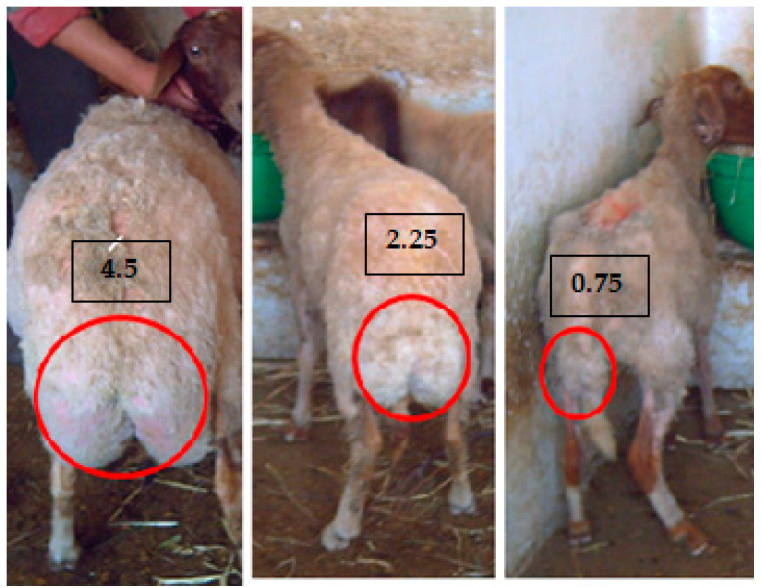
Body reserve classes according to tail size in Barbarine ewes.

For both years and breeds, one week before the introduction of rams, all ewes were weighed and scored for BCS according to Russel et al. [19]. For fat-tail BB, another BCS was accomplished at the caudal level according to Atti and Bocquier [22]; this method is more suitable to the fat-tailed breed (Figure 2). Both BCSs were graded on a five-point scale from 0 (emaciated) to 5 (obese) with divisions of 0.25 points at each score.

The condition scoring in the lumbar level was assessed by palpation to evaluate the prominence of the spinous processes in the anterior lumbar vertebrae. This evaluation involves assessing the sharpness and cover degree of the ends of the transverse processes, as well as the extent of muscular and fatty tissues beneath them. This is accomplished by spanning the lumbar vertebrae with the fingers and thumb [19]. The body condition scoring in the caudal region is also assessed by palpation in the caudal region as described by Atti and Bocquier [22]. 

Lumbar and caudal measurements were carried out by two experienced technicians and the average score value was recorded. Apart from early mating, the BW measurement and BCS assessment were periodically recorded during both years in different physiological stages including pregnancy (August), lambing (October), suckling (January), and weaning (March–April). For the Barbarine breed, the mean of the scores at the lumbar and caudal levels was considered to characterize groups. 

For lambs, the birth date, birth weight (BiW), sex, and birth type were recorded. The lambs were kept with their dams and were weighed every month until weaning at 6 months old.

### 2.4. Reproduction and Growth Parameters Calculation

The reproduction parameters were as follows:

Fertility rate = 100 ∗ Number of lambed ewes/Number of mated ewes(1)

Prolificacy rate =100 ∗ Number of born lambs/Number of lambed ewes(2)

Growth parameters were calculated by extrapolation from regular lambs’ weighing using the birth weight to estimate weights at 30 and 70 days (W_30_ and W_70_), and the average daily gains (ADG) between them (ADG0–30, ADG30–70).

### 2.5. Statistical Analyses

Ewes were regrouped in different classes according to the BW and BCS at various physiological stages to test their effects on different parameters. Statistical analyses were performed by ANOVA (analysis of variance) using the General Linear Model of SAS [23] to test the effects of breed, year, BW, and BCS at mating, lambing, and weaning on reproduction parameters and on lambs’ growth performances. Lambs’ sex and birth type effects on growth parameters were also studied. Differences among means were compared using Duncan’s multiple range test (DMRT). Correlations between all parameters were calculated through the correlation procedure of SAS. For all tests, the significance was declared at *p* < 0.05.

The multiple regression analysis of SAS was also performed to describe the relationship between lambs’ growth parameters and the variations in the BW and BCS of ewes between lambing and weaning according to the following equation:

Y = β_0_ + b_1×1_ + b_2×2_ + b_3×3_ + b_4×4_ + b_5×5_ + b_6×6_ + ε (3)

where Y is the dependent variable to predict; β_0_ is the intercept; b_n_ is the coefficient of the explanatory variable; X_n_ is the independent variable(s) used to predict or associate with Y; and ε is the regression residual error.

## 3. Results and Discussion

### 3.1. Body Weight and Body Condition Score Evolution during Two Consecutive Production Cycles

The BW and BCS of ewes at different physiological stages (mating, lambing, and weaning) as well as the variations among them according to breed and year are shown in Table 1. 

As ewes were conducted under similar conditions, ewes of both breeds started mating with similar BWs and BCSs (Table 1), averaging approximately 45 ± 5.5 kg (*p* = 0.1) and 1.89 ± 0.7 (*p* = 0.17). They also reached lambing with similar BWs (43 ± 5.1 kg, *p* = 0.06) and comparable BCSs averaging 1.71 (*p* = 0.57). At weaning, both breeds had similar BW, but a slightly higher BCS (*p* = 0.001) occurred for BB ewes compared to QFO ones (1.58 ± 0.63 vs. 1.42 ± 0.54). Between mating and lambing, the BB ewes drew more reserves compared to the QFO ones; however, among lambing and weaning, the QFO group mobilized significantly more reserves than the BB one (*p* = 0.001). Among both years, no differences were recorded for BW at mating. However, in the second year, a decline in the BCS was clearly observed (2.01 vs. 1.78), which could be explained by the lack of rain, which did not exceed 250 mm, which consequently affected pasture and the food availability. Hence, ewes lost approximately 12% (*p* < 0.05) of their BCS compared to the first year. Atti et al. [1] and González-García et al. [16] have observed similar year-dependent effects on the BCS at mating in spring. In addition, at lambing and for both breeds, ewes’ BR loss was approximately 11% of their BCS compared to the first year. These variations explain the higher reserve mobilization in order to face difficult conditions and to cover their conception needs and successfully raise offspring. 

**Figure 3:**
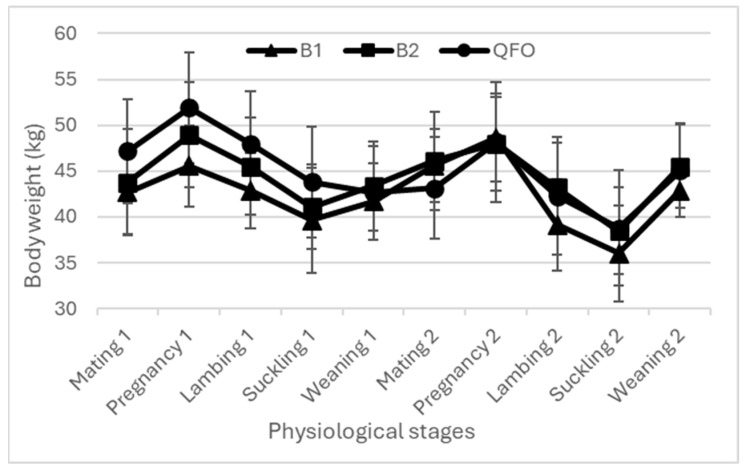
Ewes’ body weight evolution among two campaigns. B1: flock 1 of the fat-tailed Barbarine breed; B2: flock 2 of the fat-tailed Barbarine breed; QFO: flock of Queue Fine de l’Ouest breed.

**Figure 4:**
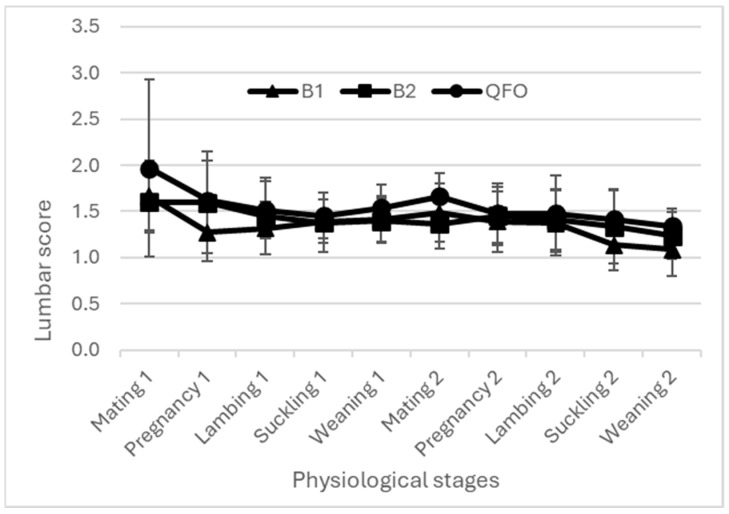
Ewes’ lumbar score evolution among two campaigns. B1: flock 1 of the fat-tailed Barbarine breed; B2: flock 2 of the fat-tailed Barbarine breed; QFO: flock of Queue Fine de l’Ouest breed.

**Figure 5:**
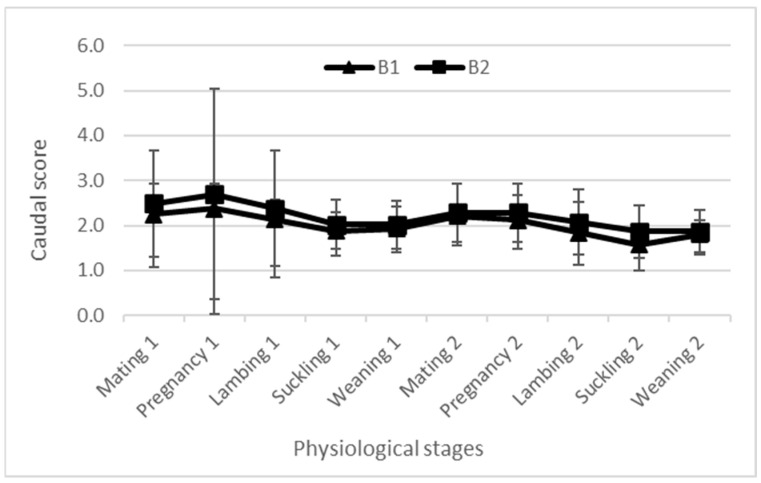
Ewes’ caudal score evolution among two campaigns. B1: flock 1 of the fat-tailed Barbarine breed; B2: flock 2 of the fat-tailed Barbarine breed.

The body weight and BCS evolutions are presented in Figure 3, Figure 4 and Figure 5. All curves present similar shapes for all flocks during both years. 

The BW and BCS were simultaneously assessed with the aim of an effective description of the dynamics of body reserves in ewes, covering both mobilization and accretion processes [2,24]. Regardless of the breed, the BW of all ewes increased during pregnancy in relationship to the anabolism of pregnancy combined with the fetus growth and development [5,25]. However, the BCS at both the lumbar and caudal levels recorded a decrease during this stage; this decrease reflects the decrease in the corporal mass of ewes and confirms the better accuracy of the BCS as a tool to assess BR. Then, BW and the BCS decreased between lambing and weaning; this drop indicates the imbalance between energy intake and the animals’ needs given the peak of milk production. A high priority given to the newborn lambs and thus dams could not meet their energy requirements [26,27]. Similar results were recorded when ewes mobilized their BR from lambing to weaning to meet the lambs’ requirements, causing low BW and BCSs [1,8,16]. After lambs’ weaning, regardless the breed, ewes’ BW and BCS increased again, given that the ewes became free from their offspring and their nutritional condition was improved [28]. This confirmed other results that explained the increase in BW and BCS during the post-weaning period due to the decrease in the ewes’ energy requirements [18,26] and dietary diversification for lambs. This is consistent with the findings of Kharrat and Bocquier [29] for goats, who reported that BR replenishment was prioritized at the end of suckling. The BR mobilization continued intensively for all breeds and for both years during the lambing and suckling stages, due to the highest energy needs induced by these physiological stages and accompanied by food scarcity. The BW and BCS profiles recorded in the current study in such conditions are consistent with the previously described dynamics between mating and weaning [1,5,16], showing the use of BR from mid-pregnancy until the end of the suckling period, which is revealed by a decrease in the BCS [5]. During lactation, irrespective of breed and lambing BCS, all ewes mobilize their BR to produce milk, even for ewes with poor BCS. The same trend as for goats was previously reported for sheep [29,30]. The BR mobilization is accentuated by low and restricted feeding, which could explain the decline of BCS at weaning in the second year of the current study, with less rainfall and thus less feed availability, where ewes weaned their lambs with poorer body conditions compared to the first campaign. 

The ewes’ BCS frequency varied from one physiological stage to another due to the BR dynamics. At mating, meagre ewes with BCS < 1.5 represented only 19% of the total ewes, which rose to 47% at lambing, while the middle-scored females with BCSs between 1.5 and 2.5 decreased from 67 to 44.8%, and only 8.8% of ewes reached lambing with a BCS higher than 2.5. The dramatic climb in the meagre ewes’ proportion vs. the fall in the proportion of middle- and well-scored ewes shows the degree of underfeeding during pregnancy and the ability of these ewes to cope with great resilience. This phenomenon continued up to weaning, where the proportion of ewes having a BCS < 1.5 continually increased to reach 56% compared to lambing and those with a BCS superior to 2.5 did not exceed 6%. These results clearly show the mobilization of BR during pregnancy, early lambing, and suckling, causing ewes to reach weaning with poor body condition scores but with weaned lambs. 

### 3.2. Reproductive Performances of Ewes 

Data on reproductive parameters (fertility and prolificacy) are shown in Table 2. 

In the current study, the fertility rates for both breeds were similar, averaging 75%, which was lower than the previously recorded results [31]. The low observed fertility rate indicates a potential shift or change in fertility patterns among these breeds, especially with climate variations and feeding resource fluctuations, which are consolidated by the slight decrease of fertility in the second year but without significant differences (*p* > 0.05). For both years, prolificacy rates were similar in both breeds and did not exceed 111%, being lower than the average value reported by the National service of Control Performances (119%). This relatively low rate of prolificacy may be in part explained by the lack of rain and the high temperatures during these two campaigns, which highly affected the pasture productivity and grass quality, and thus resulted in poor dietary behavior. In fact, ewes properly fed before and during the mating period will be relatively more prolific than underfed and leaner ewes [1]. It was reported that under the influence of climate changes or of the quantity and quality of grazed forage, supplementary feeding prior to the mating period could improve reproduction traits such as fertility and prolificacy [32]. 

The BW at mating (Table 2) significantly affected fertility but not the prolificacy rates. The lowest fertility (65%) was attributed to the lighter ewes weighing less than 40 kg. However, a regular fertility rate improvement, with a body weight increase, was observed to reach 80% for ewes weighing more than 50 kg at mating. Similar results were recorded for these breeds or for other breeds, confirming that the fertility rate increases with body weight increases [33]. Fertility was also highly affected by BCS at mating (Table 2). Ewes with poor BCS inferior to 1.5 had a significantly lower fertility rate averaging 58%, compared to ewes with a BCS between 1.5 and 2.5 and even for those exceeding 2.5 (75–80%), as previously shown [34]. 

Indeed, the positive effects of higher BW and BCSs around mating on the lambing parameters of various sheep breeds have been reported in different production systems [8,35]. Thus, the low fertility rate could be the consequence of ewes’ BW or BCS that are too low to respond to the ram effect with the prevention of estrus and fertility [36]. 

Ewes having a low BCS have higher prenatal and neonatal mortalities [37] and a lower survival rate. Similar results were found, suggesting that the poor BCS caused by a decrease in nutrient uptake induces sub-nutrition, diminishing the endometrial sensitivity to progesterone and affecting embryo survival [32]. Generally, a greater BCS at mating leads to an increased probability to manifest estrus and a higher ovulation rate, and subsequently, to a higher percentage of lambing potential [32,34]. Many researchers have reported that fertility is affected by BCS, and higher fertility rates were recorded for females with BCSs between 2 and 4 [38]. Different results accomplished in different regions on various genotypes were recorded [34]. The highest prolificacy rate was recorded for ewes whose BCS at mating was superior to 2.5, reaching 115% but without significant differences. However, it was reported that the BCS had a significant effect on litter size [20,24].

In this study, the fertility rate was positively correlated with the BW and BCS at mating and the correlations were highly significant (Table 3). 

Indeed, many reports suggested a positive correlation between the BCS and reproductive performance [8,34]. The estimation of BR at mating by means of BW or the BCS has demonstrated the existence of a positive relation to ovulation rate and fertility, or to a lesser extent, the prolificacy of several sheep breeds [1,26]. However, fertility was negatively correlated with BW and the BCS at lambing but the correlations were low and significant. Likewise, the fertility was correlated with variations in BW (r = 37) and BCS (r = 40) between mating and lambing with highly significant correlations. Prolificacy was positively correlated with BW at mating but negatively correlated with BCS at mating. Prolificacy was negatively correlated with BW and the BCS at lambing and both correlations were low and insignificant. However, the prolificacy was strongly and positively correlated with the variation in the BCS between mating and lambing.

**Figure 6:**
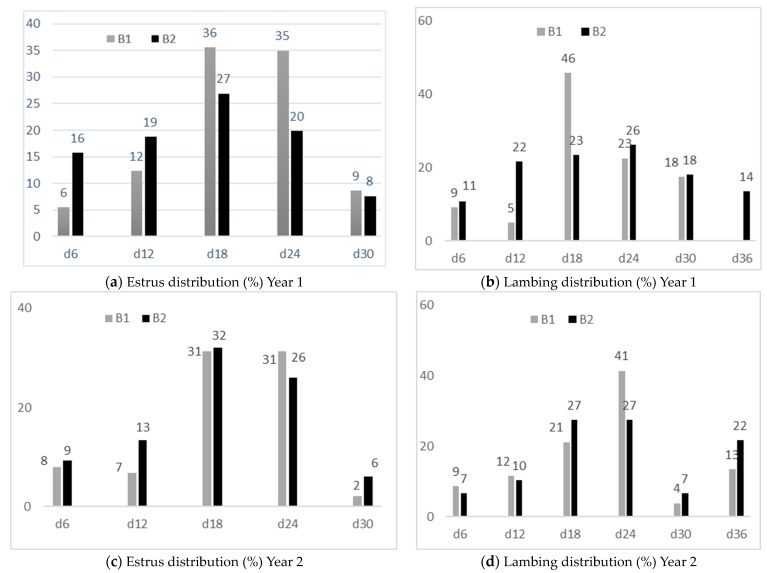
Estrus and lambing distribution over two years. B1: flock 1 of the fat-tailed Barbarine breed; B2: flock 2 the of fat-tailed Barbarine breed.

For both fat-tailed Barbarine flocks, the estrus and lambing frequency during both years are shown in Figure 6. 

Before male introduction, all ewes were anovulatory; in the first year, the proportions of ewes that had a normal induced cycle (up to 18 days) were 54 and 62% for B1 and B2, respectively. However, for the second year, these proportions were only 46 and 54% for B1 and B2, respectively. Another estrus wave appeared around the 24th day, and then these ewes displayed a short induced cycle followed by a normal cycle [39] averaging 27% and 28% for year 1 and year 2, respectively. Beyond the 24th day, for both years, a low proportion of females manifesting estrus behavior was observed; it did not exceed 10 and 5% for year 1 and year 2, respectively. Hence, one month after the rams’ introduction, approximately 98 and 90% of ewes for B1 and B2, respectively, had shown estrus behavior. Then, the rate of return to estrus was low, averaging 7%. However, in the second year, only 79 and 86% of ewes from B1 and B2, respectively, showed estrus behavior, with an average decrease of 10% in comparison to the first year. This result is clearly due to the poorer nutrition and thus lower BCS of ewes in the second year. It was reported that for ewes with low BCSs, the absence or attenuation of estrus can occur, and females with higher BCSs had a higher probability to manifest estrus [24]. The lambing distribution showed a total of 83 and 82% for B1 and B2, respectively, up to 24 days from the beginning of lambing in the first year. However, in the second year, 83 and 71% of ewes from B1 and B2, respectively, lambed within the first 24 days of lambing.

### 3.3. Offspring Growth Performances

Lambs’ growth parameters are shown in Table 4. 

Despite the difference among breeds (fat-tailed vs. thin-tailed), they drew similarly upon their reserves to give birth to lambs with the same birth weights (Bi-W) and similar growth parameters. However, in other studies, it has been reported that the breed significantly influenced the post-natal lambs’ growth in relationship with the own genotype effect, which manifests when lambs become independent of maternal effects [40]. The lambs’ birth weight was significantly lower in the second year (*p* < 0.05). The effect of the year and the lambing season, in grazing systems, on the lambs’ birth weight was previously recorded [41]. Lambs’ post-natal growth depends on the birth weight, the milk production of ewes, and the rate of introduction of solid food into their diet. Given the food shortage in the second year, milk production was presumably affected and consequently the lambs’ growth during all growth periods was also affected, which explains the inferior weights and ADG in the second year. The sex did not affect the lambs’ birth weight, which confirms other results [42]. However, at 30 and 70 days of age, males were heavier than females and the same tendency was recorded for both ADG. The lambs’ sex’s effect on the birth and advanced weights was previously reported, where male lambs were heavier than females [16,40]. The birth type significantly affected birth weight, weights at 30 and 70 days, and both ADG. In fact, single-born lambs were heavier than multiple-born ones (*p* < 0.001). This result corroborates other results [16,42] suggesting that the birth type significantly affects the birth weight. 

The BCS at lambing did not affect lambs’ Bi-W; it was comparable for lambs of both breeds and years, averaging 3.81 kg. Similar results were recorded for the same breed [28] and other breeds [42]. However, in others works, the ewes’ BCS did not highly affect the total lamb birth weight and lamb weaning weight [18]. It is clear that thin ewes have mobilized their BR during pregnancy to respond to the pregnancy needs [25]. In the current study, ewes with the highest BCS at lambing produced lambs with higher birth weights compared to ewes with low BCSs [32]. It was suggested that when ewes are malnourished throughout the final phase of pregnancy, negative effects can occur, especially during the postpartum moment, leading to less milk production with further negative consequences on the development of their lambs [43].

Both weights (W30 and W70) and both ADG were significantly higher for ewes with BCSs at lambing superior to 2; however, ewes with lower BCSs birthed lighter lambs. A positive relationship among ewes’ BCSs and lambs’ growth was reported [44]. These differences could be the consequence of a higher milk production, due to an intense BR mobilization of ewes both under better body conditions and under undernutrition conditions, causing them to produce more milk to lactate their lambs [25]. Indeed, some works recorded a significant effect of the BCS at lambing on the lamb birth weight and at the ages of 30, 60, 90, and 120 days [45]. However, other ones reported no effect [20,33,42]. This variation is probably the consequence of the timing of the BCS measurement, the ewes’ nutrition and specific breeds’ characteristics. It was shown that the BW and BCS of ewes with a maternal ability for lamb growth and/or high genetic merit would drop during suckling, especially at the beginning; the mobilized BR would be used in milk production. Nevertheless, these animals have the capacity to reconstitute new BR during other stages [8,28,35]. All growth parameters were significantly affected by BCS at weaning; the higher values were recorded for lambs issued from ewes with BCSs superior to 1.5. For all lambs, both ADG were almost similar. 

Negative and significant correlations (*p* < 0.01) were established between the BW and BCS at lambing and the birth weight (Table 5). Hence, ewes with an ability to mobilize BR during pregnancy reached lambing with low BCSs but gave birth to heavier lambs than those that did not mobilize BR. Both weights at the ages of 30 and 70 days were positively correlated with the changes in the BW and BCS between lambing and weaning, especially for W30. This growth parameter was strongly correlated with BW and BCS variations; the high coefficient of the regression analysis (Table 6) explains these correlations (0.91 and 0.78 for W30 and W70, respectively).

Both ADG showed a significant negative correlation with BW at weaning; however, they were positively correlated with the variation in BW between lambing and weaning. Additionally, the robustness of this result is emphasized by high regression coefficients (Table 6) exceeding 0.9 (0.98 and 0.95 for ADG0–30 and ADG30–70, respectively). These results also confirm the behavior of resilient ewes, which continually mobilize their BR to achieve better growth of their offspring than other ewes, reaching weaning in a poor body condition. A moderately positive correlation (r = 0.26) between ADG0–30 and BCS at weaning was recorded, while ADG30–70 was negatively correlated with this parameter and the correlation was highly significant (r = −0.91). However, in earlier results, positive and significant correlations between all growth parameters (BW and ADG) and BW/BCS at lambing were recorded [25,44]. The correlations of both daily gains with the BCS variation were not significant (Table 5). By contrast, strong positive genetic correlations between ewes’ BR (BW or BCS) and lambs’ growth parameters were reported [4,5,35].

## 4. Conclusions

In conclusion, the current study emphasizes the importance of phenotypic measurements in defining biological properties and improving herd management. The body condition score (BCS) is crucial for assessing ewes’ fat reserves and understanding the relationship between feeding and animal performances. Maternal nutrition is key from pre-mating to late pregnancy for optimizing ewe preparation and providing higher lamb performances. Despite differences among breeds, we found a lack of variation in all traits, which underlines ewes’ similar ability to better manage their body reserves, more or less efficiently, when facing alternating situations. These findings offer a significant tool for studying and enhancing sheep robustness, laying the groundwork for a future genetic improvement program targeting BR mobilization and BCS changes in sheep.

## Figures and Tables

**Table 1:** Ewes’ body weight and body condition score variations from mating to weaning according to breed and year.

	Breed			Year		
	BB	QFO	*p*-Value	1	2	*p*-Value
BWM	44.4	45.4	0.1	44.6	44.9	0.34
BCSM	1.92	1.86	0.17	2.01 ^a^	1.78 ^b^	0.001
BWL	43.6	43.1	0.06	42.9	42.7	0.1
BCSL	1.66	1.76	0.57	1.78	1.59	0.24
BWW	41.4	41.9	0.54	41.6 ^a^	40.0 ^b^	0.04
BCSW	1.58 ^a^	1.42 ^b^	0.001	1.61 ^a^	1.49 ^b^	0.001
dBW1	−0.8 ^b^	−2.3 ^a^	0.01	−1.7 ^b^	−2.2 ^a^	0.03
dBCS1	−0.26 ^a^	−0.1 ^b^	0.03	−0.23	−0.19	0.08
dBW2	−2.2 ^a^	−1.2 ^b^	0.01	−1.3 ^b^	−2.7 ^a^	0.02
dBCS2	−0.08 ^b^	−0.34 ^a^	0.001	−0.17	−0.1	0.1

BWM: body weight at mating; BCSM: body condition score at mating; BWL: body weight at lambing; BCSL: body condition score at lambing; BWW: body weight at weaning; BCSW: body condition score at weaning; dBW1: variation of body weight between mating and lambing; dBCS1: variation of body condition score between mating and lambing; dBW2: variation of body weight between lambing and weaning; dBCS2: variation of body condition score between lambing and weaning. ^a,b^: different letters within the same row differ significantly (*p* < 0.05),

**Table 2:** Reproductive parameters of fat-tailed Barbarine (BB) and Queue Fine de l’Ouest Ewes (QFO) breeds.

		Fertility (%)	Prolificacy (%)
Breed	BB	74	110
	QFO	75	113
	*p*-value	0.43	0.41
Year	1	76	111
	2	72	111
	*p*-value	0.53	0.64
BW—Mating	BW ≤ 40 (23.4%)	65 ^b^	106
	40 < BW < 50 (59.3%)	76 ^a^	111
	BW ≥ 50 (17.3%)	80 ^a^	115
	*p*-value	0.002	0.08
BCS—Mating	BCS < 1.5 (18.7%)	58 ^b^	112
	1.5 ≤ BCS < 2 (43.7%)	77 ^a^	111
	2 ≤ BCS < 2.5 (23.9%)	80 ^a^	108
	BCS ≥ 2.5 (13.7%)	75 ^a^	115
	*p*-value	0.001	0.5

BW: body weight; BCS: body condition score; ^a,b^: different letters within the same column differ significantly (*p* < 0.05).

**Table 3:** Correlation among ewes’ BW and BCS at mating and at lambing and reproductive parameters.

	BWM	BCSM	BWL	BCSL	dBW	dBCS	Fertility	Prolificacy
BWM	1.0	0.31	0.62	0.38	−0.32	−0.10	0.22	0.09
*p*		<0.0001	<0.001	<0.0001	<0.0001	0.006	<0.0001	0.01
BCSM		1.0	−0.28	0.21	0.01	0.07	0.97	−0.11
*p*			<0.0001	<0.0001	0.69	0.04	<0.0001	0.23
BWL			1.0	0.41	0.48	0.07	−0.16	−0.04
*p*				<0.0001	<0.0001	0.04	<0.0001	0.29
BCSL				1.0	0.13	0.21	−0.08	−0.04
*p*					0.0003	<0.0001	0.01	0.28
dBW					1.0	0.29	0.05	0.04
*p*						<0.0001	0.17	0.31
dBCS						1.0	0.37	0.40
*p*							<0.0001	<0.0001
Fertility							1.0	0.17
*p*								0.001
Prolificacy								1.0

BWM: body weight at mating; BCSM: body condition score at mating; BWL: body weight at lambing; BCSL: body condition score at lambing; dBW: variation of body weight between mating and lambing; dBCS: variation of body condition score between mating and lambing.

**Table 4:** Offspring growth parameters.

		Bi-W	W30	W70	ADG0–30	ADG30–70
Breed	BB	3.78	9.33	15.4	166	176
	QFO	3.79	9.82	16.04	178	182
	*p*-value	0.62	0.1	0.3	0.29	0.35
Year	1	4.07 ^a^	9.8 ^a^	17.52 ^a^	191 ^a^	193 ^a^
	2	3.46 ^b^	7.9 ^b^	14.42 ^b^	148 ^b^	163 ^b^
	*p*-value	0.001	0.001	0.001	0.03	0.02
Sex	Male	3.43	8.29 ^a^	15.13 ^a^	162 ^a^	171 ^a^
	Female	3.38	7.85 ^b^	14.17 ^b^	149 ^b^	158 ^b^
	*p*-value	0.15	0.003	0.001	0.01	0.003
Birth type	Single	3.57 ^a^	8.61 ^a^	15.57 ^a^	168 ^a^	174 ^a^
	Twin	2.72 ^b^	5.84 ^b^	10.76 ^b^	104 ^b^	123 ^b^
	*p*-value	0.001	0.001	0.001	0.001	0.001
BCS-Lambing	BCS < 1.5 (46.6)	3.79	8.41 ^b^	15.21 ^b^	154 ^b^	170 ^b^
	1.5 < BCS < 2 (28.2)	3.73	8.86 ^b^	15.94 ^b^	171 ^ab^	177 ^b^
	2 ≤ BCS < 2.5 (16.4)	3.78	9.75 ^a^	17.55 ^a^	199 ^a^	195 ^a^
	BCS > 2.5 (8.8)	3.95	9.65 ^a^	17.21 ^a^	190 ^a^	189 ^a^
	*p*-value	0.07	0.001	0.001	0.01	0.01
BCS-Weaning	BCS < 1.5 (56.1)	3.74 ^b^	8.57 ^b^	15.45 ^b^	161 ^b^	172 ^b^
	1.5 ≤ BCS < 2 (29.8)	3.83 ^b^	9.29 ^a^	16.61 a^b^	182 ^a^	183 ^b^
	2 < BCS < 2.5 (8.2)	3.86 ^b^	9.62 ^a^	17.62 ^a^	192 ^a^	200 ^a^
	BCS ≥ 2.5 (5.9)	4.1 ^a^	9.35 ^a^	16.0 ^ab^	175 ^b^	165 ^b^
	*p*-value	0.01	0.001	0.001	0.001	0.03

Bi-W: birth weight; W30: weight at 30 days; W70: weight at 70 days; ADG0–30: average daily gain between birth and 30 days of age; ADG30–70: average daily gain between 30 and 70 days of age. ^a,b^: different letters within the same column differ significantly (*p* < 0.05).

**Table 5:** Correlation among ewes’ BW and BCS at lambing and at weaning and lambs’ growth parameters.

	BCSL	BWW	BCSW	dBW	dBCS	Lamb Bi-W	W30	W70	ADG0–30	ADG30–70
BWL	−0.16	0.11	0.16	−0.49	−0.29	−0.17	0.01	−0.13	0.04	−0.32
*p*	0.0001	0.005	0.0001	<0.0001	<0.0001	0.0006	0.87	0.01	0.40	<0.0001
BCSL	1.0	0.25	0.14	−0.37	−0.33	−0.13	−0.17	−0.01	−0.05	−0.20
*p*		<0.0001	0.0004	<0.0001	<0.0001	0.008	0.002	0.78	0.29	<0.0001
BWW		1.0	0.43	−0.31	−0.22	−0.04	−0.16	−0.15	−0.67	−0.78
*p*			<0.0001	<0.0001	<0.0001	0.43	0.002	0.005	<0.0001	<0.0001
BCSW			1.0	−0.12	0.09	0.07	0.18	0.10	0.26	−0.91
*p*				0.005	0.04	0.18	0.001	0.04	<0.0001	0.0001
dBW				1.0	0.46	0.55	0.50	0.11	0.31	0.23
*p*					<0.0001	<0.0001	<0.0001	0.05	<0.0001	<0.0001
dBCS					1.0	0.34	0.86	0.03	0.02	−0.09
*p*						<0.0001	<0.0001	0.53	0.77	0.10
Lamb Bi-W						1.0	0.89	−0.002	−0.013	−0.05
*p*							<0.0001	0.96	0.80	0.38
W30							1.0	0.10	−0.08	−0.25
*p*								0.07	0.12	<0.0001
W70								1.0	0.26	−0.17
*p*									<0.0001	0.001
ADG0–30									1.0	0.007
*p*										0.89
ADG30–70										1.0

BWL: body weight at lambing; BCSL: body condition score at lambing; BWW: body weight at weaning; BCSW: body condition score at weaning; dBW: variation of body weight between lambing and weaning; dBCS: variation of body condition score between lambing and weaning; Bi-W: birth weight; W30: weight at 30 days; W70: weight at 70 days; ADG0–30: average daily gain between birth and 30 days of age; ADG30–70: average daily gain between 30 and 70 days of age.

**Table 6:** Regression analysis of lambs’ growth parameters.

Variable	Coefficients	Standard Error	*p*-Value
W_30_; R^2^ = 0.91			
Intercept	−20.4	5.65	0.001
BWL	1.67	0.12	0.0001
BCSL	3.89	1.32	0.004
BWW	−1.19	0.06	0.0001
BCSW	1.52	0.04	0.0001
dBW	1.21	0.018	0.0001
dBCS	6.87	0.58	0.0001
W_70_; R^2^ = 0.78			
Intercept	−73.0	20.03	0.0003
BWL	0.07	0.45	0.86
BCSL	21.0	4.81	0.0001
BWW	1.28	0.25	0.0001
BCSW	−2.012	0.15	0.0001
dBW	−1.17	0.67	0.08
dBCS	28.0	2.05	0.0001
ADG0–30; R^2^ = 0.98			
Intercept	797.5	517.1	0.12
BWL	484.0	11.8	0.0001
BCSL	139.5	123.3	0.25
BWW	−521.27	6.4	0.0001
BCSW	487.7	3.9	0.0001
dBW	494.2	17.0	0.0001
dBCS	42.8	53.8	0.42
ADG30–70; R^2^ = 0.95			
Intercept	30,450	2849.8	0.0001
BWL	−8999.2	702.7	0.0001
BCSL	2235.4	732.4	0.003
BWW	8577.2	722.5	0.0001
BCSW	−8896.6	690.1	0.0001
dBW	−8707.3	707.7	0.0001
dBCS	2488.9	397.6	0.0001

BWL: body weight at lambing; BCSL: body condition score at lambing; BWW: body weight at weaning; BCSW: body condition score at weaning; dBW: variation of body weight between lambing and weaning; dBCS: variation of body condition score between lambing and weaning; W30: weight at 30 days; W70: weight at 70 days; ADG0–30: average daily gain between birth and 30 days of age; ADG30–70: average daily gain between 30 and 70 days of age; R2: determination coefficient.

## Data Availability

Data are available upon request.
